# Oral and dental manifestations of celiac disease in children: a case–control study

**DOI:** 10.1186/s12903-021-01976-4

**Published:** 2021-12-29

**Authors:** Farah A. Alsadat, Najlaa M. Alamoudi, Azza A. El-Housseiny, Osama M. Felemban, Faisal M. Dardeer, Omar I. Saadah

**Affiliations:** 1 Dental Department, Jubail General Hospital, Ministry of Health, P.O. Box 1275, Al Jubail, 31951 Saudi Arabia; 2 Pediatric Dentistry Department, Faculty of Dentistry, King Abdulaziz University, Jeddah, Saudi Arabia; 3 Pediatric Dentistry Department, Faculty of Dentistry, Alexandria University, Alexandria, Egypt; 4 Department of Pediatrics, Faculty of Medicine, King Abdulaziz University, Jeddah, Saudi Arabia; 5 Pediatric Gastroenterology Unit, Department of Pediatrics, King Abdulaziz University Hospital, Jeddah, Saudi Arabia

**Keywords:** Celiac disease, Children, Dental enamel defect, Dental caries, And aphthous ulcer

## Abstract

**Background:**

Celiac disease (CD) is an immune-mediated enteropathy. CD may also involve complications with the oral cavity, which can result in various dental and oral pathologies. There are currently a limited number of studies on the oral manifestation of CD. This study aims to compare the oral manifestations of children with CD against healthy controls in Saudi Arabia.

**Materials and methods:**

This study includes 208 children aged 6–14 years, distributed equally into CD patients and healthy controls. A parent completed and validated the interview questionnaire, which included the child's personal information and medical history. A dental examination was undertaken to measure possible recurrent aphthous stomatitis (RAS), dental enamel defects (DEDs), dental caries experience, and dental malocclusion. Data were analyzed using descriptive statistics and bivariate and multivariate analysis.

**Results:**

Two hundred and eight participants were included (104 CD patients and 104 controls). The mean age for CD patients was 10.67 ± 2.39 years and 10.69 ± 2.36 for the healthy controls. CD children had more RAS than controls (42.3% vs. 15.4%, *P* < 0.001) (OR = 4.03, 95% CI = 2.09–7.81) and more DEDs than healthy controls (70.2% vs. 34.6%, *P* < 0.001) (OR = 4.45, 95% CI = 2.48–7.97). No significant difference was found in the frequency of malocclusion between cases and controls.

**Conclusion:**

Saudi Arabian children with CD had a greater number of clinical findings of RAS and DEDs than healthy controls. Pediatric dentists should consider the possibility of CD in child patients presenting with RAS or DEDs.

## Introduction

Celiac disease (CD) is an immune related enteropathy in which small intestinal mucosal damage occurs in response to dietary gluten and other environmental triggers in individuals with genetic susceptibility; in particular, individuals carrying the HLA class II DQ2 and DQ8 haplotypes are at greatest risk [1].

The possible clinical presentations of CD can be categorized into intestinal and extraintestinal manifestations. The classical intestinal manifestations include diarrhea, abdominal pain, abdominal distension, poor weight gain, and malnutrition [2]. The non-classical (extraintestinal) manifestations include short stature, osteopenia, osteoporosis, arthritis, dermatitis herpetiformis, and a number of other autoimmune diseases [3]. Oral manifestations have also been described by patients with CD [4, 5]. Various oral conditions have been reported in the literature, including recurrent aphthous stomatitis (RAS), dental enamel defects (DEDs), and delayed dental maturity [4, 6–9].

RAS is usually described as an oval or rounded painful lesions, with well-defined margins that are surrounded by a reddish base. It can affect all pediatric age groups, and mostly involves the labial mucosa or the lateral surface of the tongue [10]. RAS has been shown to be a risk indicator for CD [11]. DEDs are defects in the quantity or quality of enamel that could involve primary or permanent dentitions. DEDs may be the only clinical sign of asymptomatic non-classical CD [3]. DEDs tend to be symmetrical on similar teeth, mostly upper and lower incisors and molars that erupt simultaneously [11, 12]. Dental caries was reported in many studies in connection with CD. However, some studies have reported inconsistent and contradictory results regarding dental caries. Some studies have reported higher prevalence in CD children than matched controls [13]. Others report a lower prevalence of dental caries in CD children than healthy controls [12, 14]. Yet some investigators have found a lack of association between dental caries and CD [7, 10, 11].

In general, understanding of CD among many health care professionals, especially dentists, is minimal, so delayed diagnosis of CD is not unusual [15]. To the best of the authors’ knowledge, there is currently no detailed and comprehensive study that examines oral features in children with CD, including soft and hard tissues, dental caries, and orthodontic needs. Thus, this study aims to assess the oral health status of children with CD with respect to RAS, DEDs, dental caries, and malocclusion in comparison with healthy controls.

## Materials and methods

### Study design and sample size calculation

This is a case–control study based on the Strengthening the Reporting of Observational Studies in Epidemiology (STROBE) [16] guidelines for reporting case–control studies. Data were collected between September 2017 and February 2020.

The sample size was calculated based on a previous study [13] using the outcome variable type I DEDs. Based on the study, the odds ratio of developing DED among CD subjects was estimated to be 3.21 compared to controls and was used for determining the total sample size, using power 80% and alpha risk 5%, which was found to be 200 participants (100 patients and 100 healthy controls).

The inclusion criteria were the following for the patient group: children aged 6–14 years with biopsy-proven clinical diagnosis of CD based on the European Society for Pediatric Gastroenterology, Hepatology, and Nutrition (ESPGHAN) criteria [1]. For the control group the inclusion criteria were as follows: healthy children with no chronic illnesses. The exclusion criteria involved children with CD that were treated with corticosteroids, had mental or physical disabilities, or congenital or chromosomal abnormalities. Participants with excessive intake of fluoride or tetracycline or with clinically manifest dental fluorosis were also excluded, as were children undergoing orthodontic treatment.

### Data collection and clinical assessment

Patients were recruited in the pediatric gastroenterology clinic at King Abdulaziz University Hospital (KAUH). One hundred forty-nine contact numbers from the list of CD patients provided by the GI department were contacted; 104 subjects responded and agreed to participate (response rate was 69.8%). In order to select a control group, each CD subject was given two invitation letters of enrolment to distribute randomly to two classmates. A total of 208 invitation letters were provided for the CD children (the response rate was 50%). If parents approved their child’s participation, a phone contact was carried out to make sure that the participants were healthy. Parents were asked to bring their child to the Pediatric Dentistry Clinics in King Abdulaziz University Dental Hospital (KAUDH). The child and parents were taken to the dental clinic, where parents were asked to complete a validated parent questionnaire. This step was followed by a clinical examination of the child. The dental exam concerned RAS and DEDs using Aine's classification system [17]. Caries experience using the decayed, missed, and filled teeth index (dmft index for primary teeth and DMFT index for permanent teeth) was carried out according to WHO criteria [18]. Oral consequences related to advanced stages of untreated caries were measured using the pulp, ulceration, fistula, or abscess index (PUFA for permenant teeth/pufa for primary teeth) [19]. Malocclusion was assessed using the Dental Aesthetic Index (DAI) [20]. The clinical examinations were performed by a single trained calibrated examiner (kappa values for intra examiner reliabilities of DMFT = 0.87, dmft = 0.85, PUFA = 0.93, pufa = 0.92, DED = 0.82, and DAI = 0.93). The parent questionnaire was formulated to assess the general health status of each child. It was written in Arabic and divided into two parts. The first part comprised information regarding the child's sociodemographic data, general medical history, medications, and specific questions concerning the diagnosis and symptoms of CD. The second part concerned knowledge of the child's dental history, dietary habits, brushing habits, and specific questions related to water sources.

### Validation and reliability of the parent questionnaire

The parent questionnaire was validated following its development. The validity was tested using content validity. It was given to a group of five medical and dental experts for the assessment of each item, based on specific criteria. Each item was given a score, and then a total average score was determined by dividing each count by the total number of experts. The content validity index for items (I-CVI) was calculated; any item with a score < 0.78 was modified in terms of structure or language. Following this, the content validity index for the whole scale (S-CVI) was calculated [21]. The mean I-CVI score was 0.96. The following content validity indices for S-CVI were obtained: relevance = 0.96, clarity = 0.97, simplicity = 0.98, and ambiguity = 0.98.

In order to test the reliability of the parent questionnaire, 20 parents of children attending KAUH not included in the study were interviewed (in the Arabic language). The interview was repeated two weeks later for test–retest reliability; the test illustrated high reliability, with an intraclass correlation (ICC) of 0.82.

### Statistical analysis

All data were revised for completeness and logical consistency, then inserted into the Statistical Package for the Social Sciences (SPSS, Inc., Chicago, I1, USA) program, version 21, for analysis. Data entry was checked twice, then checking for duplicates was performed by SPSS, followed by examining each numerical variable for outliers using dot plot, and outliers were verified. Descriptive statistics were used to examine sample characteristics; continuous variables were summarized as means and standard deviations; and counts and percentages were used to categorical variables. To explore the association between CD and the primary outcomes (i.e., DMFT/dmft, PUFA/pufa, RAS, DEDs, and malocclusion), bivariate analyses were conducted with chi-squared tests, Fisher's exact tests, independent *t*-tests, or analysis of variance (ANOVA) tests. Post-hoc analysis using Tukey correction was used in cases of statistically significant results in ANOVA tests. Following this, a multivariate analysis was carried out (as multiple logistic regression models) to predict the study outcomes, which were used to control for possible confounding between CD and other covariates. The choice of covariates in the models was based on the significance level in the bivariate analysis, i.e., all covariates that showed a significant association with the outcome were included in the model.

### Ethical considerations

Ethical considerations were followed according to the Declaration of Helsinki. Approval to execute the study was obtained from the Research Ethics Committee at King Abdulaziz University (KAU) (number 078–09-17). Also, parents had to sign a parental consent form for the participation of their child.

## Results

This case–control study comprised 208 children, aged 6–14 years, divided equally into 104 patients in the case group and 104 healthy children in the control group, all of which satisfied the inclusion and exclusion criteria.

### Baseline characteristics of the participants

Socio-demographic data revealed a mean age of 10.67 ± 2.39 years for the children with CD and 10.69 ± 2.36 years for the healthy controls (*P* = 0.971). In both the CD and control groups, 50% of participants were girls. Comparing the CD patients and control groups, there were no statistically significant differences between the two groups in terms of gender (*P* = 1.00), age categories (*P* = 0.980), family income (*P* = 0.874), mother education (*P* = 0.052), and father education (*P* = 0.781).

The oral hygiene and dietary habits of the participants are shown in Table 1. The proportion of children with CD that habitually took part in daily tooth brushing was significantly higher than the proportion of the control group (60.6% vs. 46.2%; *P* = 0.037). There were no statistically significant differences between CD patients and the controls in terms of frequency of tooth brushing (*P* = 0.535), getting help with brushing (*P* = 0.889), swallowing toothpaste (*P* = 0.42), water source (*P* = 0.249), use of Zamzam holy water (*P* = 0.507), consumption of sweets (*P* = 0.323), intake of carbohydrates (*P* = 0.308), and daily intake of soft drinks (*P* = 0.369).

**Table 1:** Oral hygiene and dietary habits among the study groups

	Celiac N = 104	Control N = 104	*P* value
	n (%)		
Brushing teeth			
Yes	63 (60.6)	48 (46.2)	0.037*^†^
No	41 (39.4)	56 (53.8)	
Daily tooth brushing^#^			
Once daily	23 (36.5)	18 (37.5)	0.535^**†**^
More than once daily	40 (63.5)	30 (62.5)	
Who brushes teeth^#^			
Child	45 (43.3)	46 (44.2)	0.889^**‡**^
Adult involved	18 (17.3)	2 (1.9)	
Swallow toothpaste (0–6 years)			
Yes	6 (5.8)	9 (8.7)	0.421^**†**^
No	98 (94.2)	95 (91.3)	
Water source (0–6 years)			
Bottle	85 (81.7)	91 (87.5)	0.249^**†**^
Tap	19 (18.3)	13 (12.5)	
Zamzam (0–6 years)			
Yes	13 (12.5)	10 (9.6)	0.507^**†**^
No	91 (87.5)	94 (90.4)	
Sweets daily intake			
< 3 times	80 (76.9)	74 (71.2)	0.343^**†**^
≥ 3 times	24 (23.1)	30 (28.8)	
Soft drinks daily intake			
< 3 times	100 (96.2)	103 (99.0)	0.369^**‡**^
≥ 3 times	4 (3.8)	1 (1.0)	
Carbohydrate daily intake			
Yes	79 (76.0)	85 (81.7)	0.308^**†**^
No	25 (24.0)	19 (18.3)	

Bold values indicate significant levels of *P* values

*N* total number of participants, *n* number of participants in each group

^#^Total number of those who brushed their teeth from the total N. *Does not brush* was not included in this analysis

^**†**^Chi-squared test

^**‡**^Fisher’s exact test was used when the number of participants is less than 5

*P < 0.05, using a chi-squared test

### Oral health status among participants

**Fig. 1:**
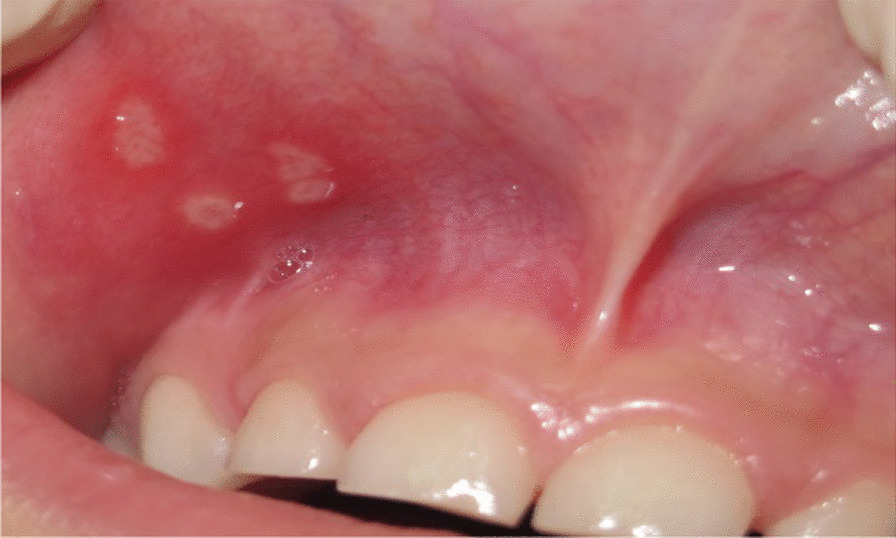
RAS**.** Three ulcerations of variable sizes in the maxillary labial mucosa of a 7-year-old girl with CD

**Fig. 2:**
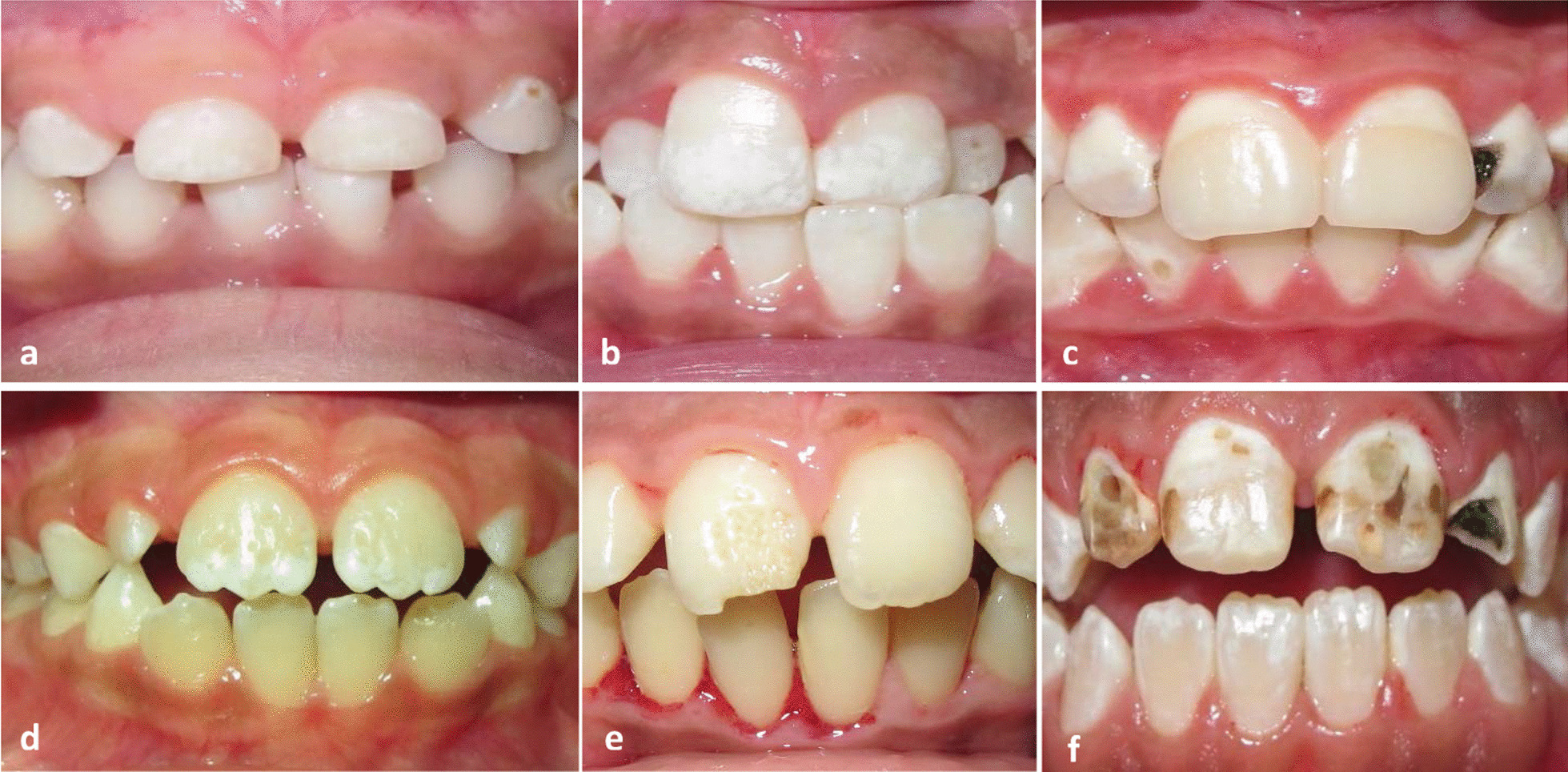
DEDs in Patients with CD. Grade I (enamel color defect): white diffuse opacities in upper incisors in primary teeth in a 7-year-old boy (**a**). Grade I (enamel color defect): white diffuse opacities in upper anterior teeth in a 10-year-old boy (**b**). Grade I (enamel color defect): clearly defined white opacities in a 13-year-old girl (**c**). Grade II (mild structural defect) shallow pits in upper central incisors in a 6.5-year-old girl (**d**). Grade III (evident structural defect): large vertical pits in a 12-year-old boy with CD (**e**). Grade III (evident structural defect): generalized large opacities of different colors, also an open bite exists in a 12-year-old girl (**f**)

In this study, RAS was found in 42.3% of CD children and 15.4% of the controls (*P* < 0.001) (Fig. 1). Having CD significantly increased the odds of having RAS by 4.03 times the rate of the healthy controls (95%, CI = 2.09–7.81). Similarly, children with CD had more frequent DEDs than controls (70.2% vs. 34.6%, *P* < 0.001), and CD children were 4.45 times more likely to have DEDs than healthy controls (95%, CI = 2.48–7.97). Figure 2a–f represents the different forms of DEDs that were recognized in the study. Furthermore, children with CD were less likely to have DEDs Grade 0 (OR = 0.27) but more likely to have DEDs Grade I (OR = 3.39) than controls. Dental caries was evaluated as caries prevalence, using the combined caries experience for both primary and permanent teeth combined (DMFT/dmft), as a dichotomous variable (DMFT/dmft > 0 or DMFT/dmft = 0). When there is no caries experience in primary or permanent teeth, the score is equal to zero; caries severity is evaluated using the continuous variable of decayed, missed, and filled index for permanent and primary teeth (DMFT/dmft). There was no statistically significant difference found between CD cases and controls in the frequency of malocclusion of various severities (Table 2).

**Table 2:** Oral health status among the CD and control groups (percentage distribution)

Variables	Celiac N = 104	Control N = 104	*P* value	OR (95% CI)
	n (%)			
RAS				
Yes	44 (42.3)	16 (15.4)	< 0.001*^†^	4.03 (2.09–7.81)
No	60 (57.7)	88 (84.6)		Ref
DED				
Yes	73 (70.2)	36 (34.6)	< 0.001*^†^	4.45 (2.48–7.97)
No	31 (29.8)	68 (65.4)		Ref
DED Grade I	51 (49.0)	23 (22.1)	< 0.001**^‡^	3.39 (1.86–6.19)
DED Grade II	18 (17.3)	11 (10.6)		1.77 (0.79–3.60)
DED > Grade II	4 (3.8)	2 (1.9)		2.04 (0.37–11.39)
Dental caries experience				
Yes	89 (85.6%)	98 (94.2%)	0.038*^†^	0.36 (0.14–0.98)
No	15 (14.4%)	6 (5.8%)		Ref
Malocclusion⁂				
No/mild	21 (21.4%)	23 (23.5%)	0.732^†^	0.89 (0.45–1.74)
Defined	28 (28.6%)	29 (29.6%)	0.875^†^	0.95 (0.51–1.76)
Severe	24 (24.5%)	21 (21.4%)	0.610^†^	1.19 (0.61–2.32)
Very severe	25 (25.5%)	25 (25.5%)	1.00^†^	1.00 (0.53–1.90)
Deep bite	13 (13.3%)	13 (13.3%)	1.00^†^	1.00 (0.44–2.28)
Posterior cross bite	13 (13.3%)	8 (8.2%)	0.248^†^	1.72 (0.68–4.36)

Bold values indicate significant levels of *P* values/OR (95% CI)

*N* total number of participants, *n* number of participants in each group, *CD* celiac disease, *OR* odds ratio, *DED Grade I* defect in enamel color, such as cream or brown opacity, well defined or diffused, *Grade II* slight defect in enamel structure as "horizontal grooves or shallow pits.”, *Grade III* evident defect in enamel structure as "deep horizontal grooves or large opacities of different colors.”, *Grade IV* severe form of defect in enamel structure as "the shape of the tooth is altered, with pointed cusps.”, *DMFT/dmft* decayed, missed, filled permanent/primary teeth

^†^Chi-squared test

^‡^Fisher’s exact test was used when the number of participants is less than 5

*P value < 0.05 using chi-squared test

**P value < 0.05 using Fisher’s exact test

⁂Patients with only primary teeth were not assessed

Dental caries experience among the study participants are shown in Table 3. Children with CD were less likely to have experienced dental caries than healthy controls. In terms of the dmft of the primary dentition, the mean dmft in children with CD (5.01 ± 4.0) was significantly lower than healthy controls (6.56 ± 4.20) (*P* = 0.019). Also, the mean number of decayed teeth in children with CD was significantly lower (3.70 ± 3.65) than healthy controls (5.13 ± 3.86) (*P* = 0.018). When considering the dental caries in the permanent dentition, DMFT showed no significant difference between the two groups (*P* = 0.200), but the mean number of decayed permanent teeth in children with CD (2.81 ± 3.21) was significantly higher than healthy controls (1.90 ± 2.18) (*P* = 0.021). Furthermore, when the dental caries consequences were analyzed as a continuous variable, the mean pufa in primary teeth was statistically lower in the CD group (1.44 ± 2.14) compared to the control group (2.66 ± 3.10) (*P* = 0.004). When the dental caries consequences were analyzed as a continuous variable, the pulpal consequences were significantly lower in children with CD (1.59 ± 2.05) than healthy controls (2.84 ± 3.19) (*P* = 0.005). Also, the pulpal consequences were significantly lower in children with CD (1.33 ± 1.93) than healthy controls (2.67 ± 3.11) (*P* = 0.001). The mean PUFA in permanent teeth was not statistically different between the two groups (*P* = 0.096) but the pulpal consequences were significantly higher in children with CD (0.46 ± 1.02) than healthy controls (0.22 ± 0.53) (*P* = 0.044).

**Table 3:** Oral health conditions among the CD and control groups (mean ± SD)

Variables	Celiac N = 104	Control N = 104	*P* value^†^
	Mean ± SD (95% CI)		
Caries experience including primary and permanent teeth			
Decayed	5.43 ± 1.05 (4.65–6.22)	5.78 ± 4.29 (4.95–6.61)	0.550
Missed	0.70 ± 1.71 (0.31–1.09)	0.34 ± 0.80 (0.15–0.52)	0.103
Filled	0.96 ± 1.83 (0.54–1.38)	1.43 ± 2.22 (0.92–1.95)	0.157

	n = 81	n = 79	
Caries experience dmft (primary teeth only)			
Decayed primary	3.70 ± 3.65 (2.92–4.52)	5.13 ± 3.86 (4.12–5.96)	0.018*
Missing primary	0.64 ± 1.67 (0.30–1.09)	0.30 ± 0.72 (0.13–0.47)	0.098
Filled primary	0.67 ± 1.49 (0.37–1.07)	1.13 ± 1.85 (0.63–1.39)	0.085
Primary dmft	5.01 ± 4.05 (4.23–6.03)	6.56 ± 4.20 (5.42–7.29)	0.019*

	n = 98	n = 98	
Caries experience DMFT (permanent teeth only)			
Decayed permanent	2.81 ± 3.21 (2.14–3.42)	1.90 ± 2.18 (1.46–2.34)	0.021*
Missing permanent	0.05 ± 0.22 (0.01–0.09)	0.06 ± 0.24 (0.01–0.11)	0.758
Filled permanent	0.34 ± 1.19 (0.10–0.57)	0.62 ± 1.62 (0.30–0.95)	0.162
Permanent DMFT	3.20 ± 3.54 (2.49–3.91)	2.62 ± 2.75 (2.07–3.17)	0.200
Caries consequences in primary and permanent teeth			
Pulpal	1.59 ± 2.05 (1.12–2.06)	2.84 ± 3.19 (2.10–3.58)	0.005*
Ulceration	0 (0.00)	0 (0.00)	–
Fistula	0.03 ± 0.16 (− 0.01–0.06)	0.04 ± 0.26 (− 0.02–0.10)	0.686
Abscess	0.08 ± 0.32 (0.01–0.15)	0.11 ± 0.31 (0.04–0.18)	0.571

	n = 81	n = 79	
Caries consequences pufa (primary teeth only)			
Pulpal primary	1.33 ± 1.93 (0.92–1.80)	2.67 ± 3.11 (1.95–3.42)	0.001*
Ulceration primary	0 (0.00)	0 (0.00)	–
Fistula primary	0.02 ± 0.15 (− 0.01–0.06)	0.04 ± 0.25 (− 0.02–0.10)	0.687
Abscess primary	0.09 ± 0.32 (0.01–0.16)	0.08 ± 0.27 (0.02–0.15)	0.824
Primary pufa total	1.44 ± 2.14 (0.98–1.95)	2.66 ± 3.10 (1.94–3.41)	0.004*

	n = 98	n = 98	
Caries consequences PUFA (permanent teeth only)			
Pulpal permanent	0.46 ± 1.02 (0.26–0.65)	0.22 ± 0.53 (0.06–0.27)	0.044*
Ulceration permanent	0 (0.00)	0 (0.00)	–
Fistula permanent	0.01 ± 0.10 (− 0.01–0.03)	0 (0.00)	0.319
Abscess permanent	0 (0.00)	0.02 ± 0.14 (− 0.01–0.04)	0.157
Permanent PUFA	0.47 ± 1.05 (0.25–0.68)	0.27 ± 0.60 (0.09–0.32)	0.096

Bold values indicate significant levels of *P* values

*N* total number of participants, *n* number of participants in each group, *CI* confidence interval, *DMFT/dmft* decayed, missed, filled permanent/primary teeth, *P* pulp, *U* ulceration, *F* fistula, *A* abscess, *SD* standard deviation

^†^Independent t-test

*P < 0.05

### Effect of CD on development of RAS, DEDs, and dental caries after adjustment for teeth brushing

Three multiple logistic regressions were used to evaluate the effects of CD on RAS, DED, and dental caries, controlling for teeth brushing. Children with CD had 3.79 times more likely of having RAS (95%, CI 1.96–7.35) compared to children without CD (*P* < 0.001). Also, the CD group were 4.39 times more likely to have DEDs (95% CI, 2.44–7.89) than controls. CD was not shown to be a significant predictor of dental caries (*P* = 0.235) in this model (Table 4).

**Table 4:** Multiple logistic regression models to evaluate the effect of CD on independent variables controlling for teeth brushing

	RAS	DED	Dental caries (yes vs. no)
	OR (95% CI) *P* value	OR (95% CI) *P* value	OR (95% CI) *P* value
Celiac disease			
Yes	3.79 (1.96–7.35) *P* < 0.001*	4.39 (2.44–7.89) *P* < 0.001*	0.55 (0.21–1.47) *P* = 0.235
No	Reference	Reference	Reference
Brushing teeth			
Yes	0.74 (0.39–1.40) *P* = 0.350	1.11 (0.62–2.00) *P* = 0.726	0.18 (0.05–0.63) *P* = 0.007*
No	Reference	Reference	Reference

Bold values indicate statistically significant *P* values

*P < 0.05

*RAS* recurrent aphthous stomatitis, *DED* dental enamel defects, *OR* odds ratio; dependent variable = CD; independent variables = RAS, DEDs and dental caries

## Discussion

Celiac disease (CD) is an autoimmune enteropathy affecting the small intestine in which the ingestion of gluten, possibly with other environmental factors, trigger damage to the intestinal villi, often leading to atrophy [1]. Oral manifestations of CD have been the focus of numerous studies. Generally, studies report on a single manifestation or multiple oral manifestations [3, 4, 7–9, 15, 22]. In the present study, CD increased the likelihood of having RAS by about 4.03 times healthy controls, in agreement with one previous study, which reported a similar odds ratio (OR = 4.12) [23]. Several studies also reported a greater occurrence of RAS in children with CD than healthy controls [11, 12, 15, 24, 25]. Only one case–control study, using a smaller sample size, failed to show an association [7]. We found also that DEDs are more prevalent in children with CD than healthy controls, where children with CD had a greater prevalence of DEDs, supporting some previously reported studies [7, 11, 12, 15, 24, 25]. Conversely, one study reported no difference in the prevalence of DEDs in CD children and controls [23].

DEDs occur during the period of teeth formation when exposure to gluten may play a role in development [12]. Gluten triggers the production of antibodies against the enamel proteins, presumably causing DEDs, either in primary or permanent teeth. These antibodies may pass through the placental blood barrier during tooth formation [26]. Thus, early diagnosis of CD at a younger age and the institution of a gluten-free diet (GFD) may prevent the development of DEDs in permanent teeth [26, 27].

Several reports of the association of dental caries in children with CD have shown inconsistent results [7, 10–14]. Our study showed that children with CD had a lower caries experience and had a lower likelihood of experiencing caries than healthy controls. This finding is supported by one study that found that dental caries in permanent teeth in a CD group was lower than controls [13]. In our study, dental caries experience was measured using DMFT/dmft scores. In primary teeth, the scores were significantly lower in the CD group than healthy controls. However, in permanent teeth, children with CD showed a significantly higher number of decayed teeth than did healthy controls. These two results correspond with a previous study which showed that in permanent teeth there were substantially higher mean DMFT scores in children with CD than controls. In contrast, in primary teeth, children with CD had lower mean dmft scores than controls, but this was statistically insignificant [24]. It should also be noted that some previous studies documented a statistically insignificant difference between children with CD and healthy controls for mean caries scores [7, 11, 25].

Perhaps the increased severity of decayed permanent teeth in the current study is related to DEDs that affect tooth structure, the lower calcium to phosphorus ratio, or the decreased salivary flow, resulting in more straightforward dissolution in CD patients [12].

Our study found that dental caries experience as well as decayed primary teeth were lower in children with CD, which may be due to good adherence to a GFD, although adherence to a GFD was not assessed in the present study. Another study found that CD children, who were compliant with a GFD, had a lower number of caries than both those that were less compliant and controls [13], which may serve to underscore the importance of implementation of a GFD. A GFD could explain the reduced dental caries in primary teeth in CD patients, because of the early exclusion of a cariogenic foods, such as grains and sweets. It is important to be cognizant of the fact that dental caries is a multifactorial condition that may concern enamel and salivary composition, including reduced flow rate, dietary factors, and oral hygiene.

In this study, to assess the caries experience, the DMFT/dmft and the PUFA/pufa indices were used, assessing diseases occurring pulpo-periapically, which could result from untreated dental caries. This index may help to understand the complete picture of caries present in an individual [19].

We did not find any difference in the occurrence of malocclusion between children with CD and healthy controls. This finding contradicts one previous study that reported a higher rate of malocclusion in CD patients than in healthy controls [28]. However, several factors may contribute to the development of malocclusion, including delayed dental eruption and mandibular growth [29].

A strength of the present study is its case–control design, which involves the presence of an adequate sample size, homogeneity of the population studied, and the combination of clinical assessment and information gathered from a validated questionnaire.

In conclusion, children with CD have higher odds of RAS and DEDs than healthy controls. Also, CD patients had a higher prevalence of dental caries in permanent teeth and a lower frequency in primary teeth.

## Data Availability

The datasets used and/or analyzed during the current study available from the corresponding author on reasonable request.

## Abbreviations

ASA: American Society of Anesthesiologists
CD: Celiac disease
CI: Confidence interval
DAI: Dental aesthetic index
DEDs: Dental enamel defects
DMFT/dmft: Decayed missed and filled permanent or primary teeth
ESPGHAN: European Society of Pediatric Gastroenterology, Hepatology and Nutrition
GFD: Gluten-free diet
ICC: Intraclass correlation
I-CVI: Content validity index for item(s)
KAU: King Abdulaziz University
KAUDH: King Abdulaziz University Dental Hospital
KAUH: King Abdulaziz University Hospital
OR: Odds ratio
PUFA: Pulp, ulceration, fistula or Abscess (an index of clinical consequences of untreated caries)
RAS: Recurrent aphthous stomatitis
S-CVI: Content validity index for scale(s)
STROBE: Strengthening The Reporting of Observational Studies in Epidemiology

## References

[1] Husby S, Koletzko S, Korponay-Szabó IR, Mearin ML, Phillips A, Shamir R (2012). European Society for Pediatric Gastroenterology, hepatology, and nutrition guidelines for the diagnosis of coeliac disease. J Pediatr Gastroenterol Nutr.

[2] Saadah OI (2011). Celiac disease in children and adolescents at a singe center in Saudi Arabia. Ann Saudi Med.

[3] van Gils T, Brand HS, de Boer NK, Mulder CJ, Bouma G (2017). Gastrointestinal diseases and their oro-dental manifestations: part 3—coeliac disease. Br Dent J.

[4] Karlin S, Karlin E, Meiller T, Bashirelahi N (2016). Dental and oral considerations in pediatric celiac disease. J Dent Child (Chic).

[5] Ouda S, Saadah O, El Meligy O, Alaki S (2010). Genetic and dental study of patients with celiac disease. J Clin Pediatr Dent.

[6] Alamoudi NM, Alsadat FA, El-Housseiny AA, Felemban OM, Al Tuwirqi AA, Mosli RH (2020). Dental maturity in children with celiac disease: a case-control study. BMC Oral Health.

[7] Cruz I-T-S-A, Fraiz F-C, Celli A, Amenabar J-M, Assunção L-R-S (2018). Dental and oral manifestations of celiac disease. Med Oral Patol Oral Cir Bucal.

[8] Macho VMP, Coelho AS, Veloso E Silva DM, de Andrade DJC (2017). Oral manifestations in pediatric patients with coeliac disease—a review article. Open Dent J.

[9] Mantegazza C, Paglia M, Angiero F, Crippa R (2016). Oral manifestations of gastrointestinal diseases in children. Part 4: coeliac disease. Eur J Paediatr Dent.

[10] Bramanti E, Cicciù M, Matacena G, Costa S, Magazzù G (2014). Clinical evaluation of specific oral manifestations in pediatric patients with ascertained versus potential coeliac disease: a cross-sectional study. Gastroenterol Res Pract.

[11] Acar S, Yetkıner AA, Ersın N, Oncag O, Aydogdu S, Arıkan C (2012). Oral findings and salivary parameters in children with celiac disease: a preliminary study. Med Princ Pract.

[12] Carvalho FK, Queiroz AM, Silva RAB (2015). Oral aspects in celiac disease children: clinical and dental enamel chemical evaluation. Oral Surg Oral Med Oral Pathol Oral Radiol.

[13] Avşar A, Kalayci AG (2008). The presence and distribution of dental enamel defects and caries in children with celiac disease. Turk J Pediatr.

[14] Maloney WJ, Raymond G, Hershkowitz D, Rochlen G (2014). Oral and dental manifestations of celiac disease. N Y State Dent J.

[15] Villemur Moreau L, Dicky O, Mas E, Noirrit E, Marty M, Vaysse F (2021). Oral manifestations of celiac disease in French children. Arch Pediatr.

[16] Vandenbroucke JP, Elm E, Altman DG (2007). Strengthening the Reporting of Observational Studies in Epidemiology (STROBE): explanation and elaboration. Epidemiology.

[17] Aine L, Mäki M, Collin P, Keyriläinen O (1990). Dental enamel defects in celiac disease. J Oral Pathol Med.

[18] WHO (2013). Oral health surveys: basic methods.

[19] Monse B, Heinrich-Weltzien R, Benzian H, Holmgren C, Palenstein HW (2010). PUFA—an index of clinical consequences of untreated dental caries. Community Dent Oral Epidemiol.

[20] Cons NC, Jenny J, Kohout FJ, Songpaisan Y, Jotikastira D (1989). Utility of the dental aesthetic index in industrialized and developing countries. J Public Health Dent.

[21] Polit DF, Beck CT (2006). The content validity index: are you sure you know what’s being reported? Critique and recommendations. Res Nurs Health.

[22] Shteyer E, Berson T, Lachmanovitz O, Hidas A, Wilschanski M, Menachem M (2013). Oral health status and salivary properties in relation to gluten-free diet in children with celiac disease. J Pediatr Gastroenterol Nutr.

[23] Procaccini M, Campisi G, Bufo P, Compilato D, Massaccesi C, Catassi C (2007). Lack of association between celiac disease and dental enamel hypoplasia in a case-control study from an Italian central region. Head Face Med.

[24] Cantekin K, Arslan D, Delikan E (2015). Presence and distribution of dental enamel defects, recurrent aphthous lesions and dental caries in children with celiac disease. Pak J Med Sci Q.

[25] Zoumpoulakis M, Fotoulaki M, Topitsoglou V, Lazidou P, Zouloumis L, Kotsanos N (2019). Prevalence of dental enamel defects, aphthous-like ulcers and other oral manifestations in celiac children and adolescents: a comparative study. J Clin Pediatr Dent.

[26] Sóñora C, Arbildi P, Rodríguez-Camejo C, Beovide V, Marco A, Hernández A (2016). Enamel organ proteins as targets for antibodies in celiac disease: implications for oral health. Eur J Oral Sci.

[27] Bossù M, Bartoli A, Orsini G, Luppino E, Polimeni A (2007). Enamel hypoplasia in coeliac children: a potential clinical marker of early diagnosis. Eur J Paediatr Dent.

[28] Abdul-Wahid SF, Al-Azawi LA, Ibrahim IT (2005). Enamel defects and malocclusion in patients with celiac disease. J Baghdad Coll Dent.

[29] Bilello G, Ciulla C, Caradonna C (2010). Celiac disease and malocclusion. Recent Prog Med.

